# Sensing Based on Plasmon-Induced Transparency in H-Shaped Graphene-Based Metamaterials

**DOI:** 10.3390/nano14120997

**Published:** 2024-06-08

**Authors:** Xiongxiong Wu, Jiani Chen, Shaolong Wang, Yang Ren, Yanning Yang, Zhihui He

**Affiliations:** School of Physics and Electronic Information, Yan’an University, Yan’an 716000, China; chenjiani202405@163.com (J.C.); wangshaolong2024@163.com (S.W.); yananry@163.com (Y.R.); hezh@yau.edu.cn (Z.H.)

**Keywords:** surface plasmon polaritons, graphene, terahertz, sensing, plasmon-induced transparency

## Abstract

Graphene can support surface plasmon polaritons (SPPs) in the terahertz band, and graphene SPP sensors are widely used in the field of terahertz micro- and nano-optical devices. In this paper, we propose an H-shaped graphene metasurface and investigate the plasmon-induced transparency (PIT) phenomenon in the proposed structure using the finite-difference time-domain (FDTD) method. Our results show that the Fermi energy levels, as well as certain shape parameters, can effectively modulate the PIT phenomenon in the proposed structure. Interestingly, changing some of these shape parameters can excite two dips into three. In terms of sensing performance, the maximum values of sensitivity and figure of merit (FOM) are 1.4028 THz/RIU and 17.97, respectively. These results offer valuable guidance for the use of terahertz optical graphene SPP sensors.

## 1. Introduction

In recent years, there has been a growing interest among researchers in exploring terahertz radiation, which has the unique ability to reveal the physical and chemical properties of matter through the absorption and reflection spectra of various substances [1,2]. The terahertz spectrum contains a vast amount of information that can be used to investigate the optical properties of different materials [3,4,5]. As a result of this, the terahertz spectrum has found applications in a range of fields, including terahertz spectroscopy for agricultural product quality testing [6,7], terahertz sensing [8], and terahertz imaging [9].

Graphene, a two-dimensional material consisting of a single layer of carbon atoms, possesses unique physical features [10,11], such as high transmission [12], high thermal conductivity [13], and room-temperature ferromagnetism [14]. At the terahertz wave band, graphene can generate SPPs that are highly localized and low-loss [15,16,17,18]. SPPs are electromagnetic waves generated by the excitation of free electrons on the surfaces of metals and media by incident light [19,20,21,22,23,24,25].

Fano resonance is a highly fascinating phenomenon in photonics systems [26,27]. As SPPs have advantages like strong enhancement of the local electric field and much better adaptability to nanoarchitectures, Fano resonance is widely achieved in the micro- and nano-optical interference domains that exploit SPPs [28,29]. In the terahertz band, when incident light interacts with a graphene surface, two different transmission modes are generated. The first mode is a superradiant resonance mode, also known as the bright mode, which exhibits broadband features and is directly excited by the incident light. The second mode is a subradiant resonance mode, also known as the dark mode, which has narrowband features and is indirectly excited. These modes interfere and couple with each other, resulting in the PIT phenomenon [30,31].

The conventional sensors that use metal metasurfaces are inefficient due to their excessive ohmic losses. However, graphene metasurface offers outstanding advantages because it has significantly lower ohmic losses [32,33], which leads researchers to propose various PIT sensing optical devices based on different structures of graphene metasurface. For example, in 2018, Tang et al. realized sensing with a sensitivity of 0.36 THz/RIU based on a graphene micro-ribbon array structure [34]. In 2020, He W. et al. designed a periodic metasurface structure consisting of wide horizontal graphene and two graphene sheets with high sensitivity [35]. In 2021, Su et al. investigated terahertz sensing with 1.17 THz/RIU in metamaterials consisting of monolayer graphene and two metal-distributed Bragg reflectors [36]. In 2022, Yixuan Wang et al. designed a periodic metasurface structure consisting of continuous graphene bands and truncated graphene bands with a sensitivity of up to 0.7928 THz/RIU [37]. In 2022, Zhenxiong Li et al. designed a metasurface structure that contains a graphene ribbon and U-shaped graphene [38]. Research in the field of graphene SPP sensors has yielded several impressive results. Nonetheless, despite the significant progress that has been made, there is still an urgent need to further increase the sensitivity of these sensors and optimize their structures.

This paper focuses on the design of an H-shaped periodic structure for graphene metasurfaces based on the PIT phenomenon. The H-shaped structure comprises a horizontal element and two vertical elements, with the horizontal component acting as the bright mode and the two vertical components acting as the dark mode. By conducting FDTD simulations, we observed that adjusting the Fermi energy levels or changing the shape parameters of the proposed H-shaped periodic graphene metasurface can effectively tune the PIT phenomenon. Importantly, by adjusting certain parameters, we can obtain three separate dips in the PIT response. Through these findings, we further explore the sensing performance of the structure and find that Dip1 exhibits a maximum sensitivity of 0.5612 THz/RIU, while Dip2 exhibits a maximum sensitivity of 1.428 THz/RIU and a maximum FOM of up to 17.97 at a resonant frequency of 6.02204 THz.

## 2. Structure and Theoretical Model

**Figure 1:**
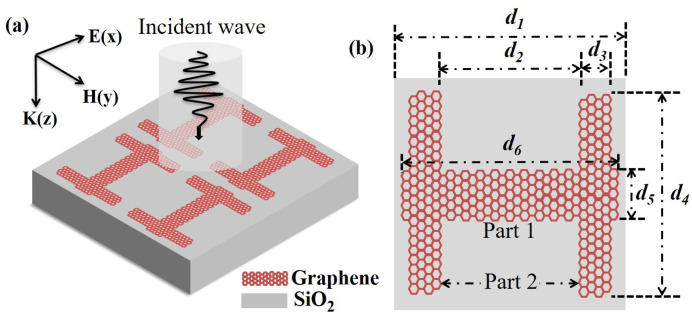
(**a**) The 3D schematic diagram of the periodic structure. (**b**) Schematic of unit cell, where *d*_1_ = 5 μm, *d*_2_ = 2.2 μm, *d*_3_ = 0.8 μm, *d*_4_ = 4.2 μm, *d*_5_ = 1.5 μm, and *d*_6_ = 4.5 μm.

Through an exhaustive literature review, our investigation into the PIT phenomenon, which relies on plasmonic excitation, revealed that achieving this effect involves two modes of destructive interference. Inspired by this finding, we designed a novel periodic structure of graphene metasurface, incorporating two vertical elements appended to a rectangular horizontal component to attain the intended effect. The structure was validated using finite-difference time-domain (FDTD) simulations, and its design is illustrated in Figure 1. The structure consists of a rectangular, square substrate and a graphene material layer, which are represented in grey and red, respectively, as shown in Figure 1. The substrate is composed of silicon dioxide, and the graphene material layer is adsorbed onto the upper surface of the substrate. Each unit cell in the structure has an H-shaped configuration.

We can achieve subjective control of conductivity by applying an electric field to the graphene material to move its Fermi surface and thus change its conductivity, which can be described by using Kubo’s formula [39,40,41,42]:

(1) $$\sigma =\frac{ie^{2}E_{f}}{\pi \hbar (\omega +i\tau^{-1})},$$

where ω, $E_{f}$, e, and ħ are the frequency of the incident wave, the Fermi energy, the electron charge, and the reduced Planck constant, respectively. τ is the carrier relaxation time, which can be expressed by $\tau =\mu E_{f}/e\cdot {\nu^{2}}_{F}$, with μ = 1 m^2^/(Vs) and $\nu_{F}$ = 10^6^ m/s.

In Equation (1), $E_{f}$ is the Fermi energy of graphene, which can be given by the following equation:

(2) $$E_{f}=\hbar v_{F}{\left(\frac{\pi \varepsilon_{0}\varepsilon_{si}V_{g}}{d_{c}e}\right)}^{\frac{1}{2}},$$

Here, $\varepsilon_{si}$, $\varepsilon_{0}$, e, $d_{c}$, $\nu_{F}$, and $V_{g}$ are the static permittivity of silicon, the vacuum permittivity, the electron charge, the silicon thickness, the Fermi velocity, and the gate voltage, respectively. According to Equations (1) and (2), the Fermi energy level of graphene can be adjusted by varying the gate voltage, enabling subjective adjustment over its conductivity.

**Figure 2:**
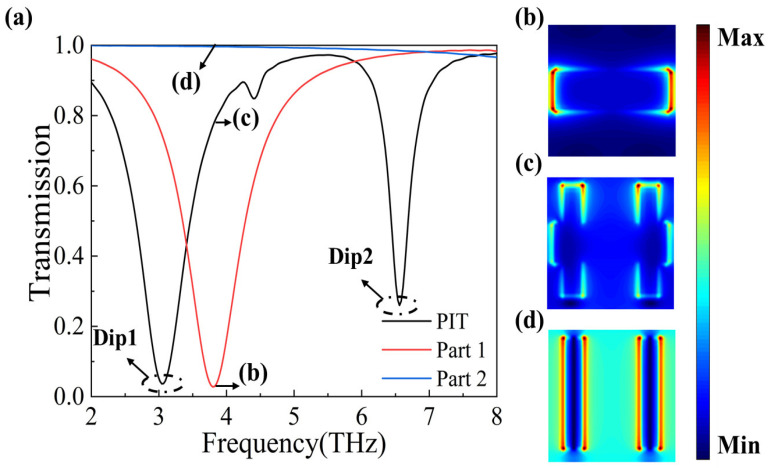
(**a**) Transmission spectra of graphene metasurface at a Fermi energy level of 1.0 eV, with the red solid line for the bright mode (Part 1), the blue solid line for the dark mode (Part 2), and the black solid line for the PIT (all parts). Figures (**b**–**d**) correspond to the electric field distributions at points (b), (c), and (d) labeled in figure (**a**), respectively.

In the simulation, we assumed that the x-axis and y-axis directions were infinitely long. The periodic boundary conditions are applied in the x-axis and y-axis directions, and perfectly matched layers are applied in the z-direction. The incident light was irradiated vertically to the graphene metamaterial surface along the z-axis, and we observed a significant PIT phenomenon. We designated the horizontal graphene component and the two vertical graphene components as Part 1 and Part 2, respectively, and then analyzed the phenomenon using the theory of bright and dark modes. As shown in Figure 2a, when only Part 1 is present, the transmission spectrum (red curve) exhibits a bright mode that is directly excited by the incident light. Conversely, when only Part 2 is present, the transmission spectrum (blue curve) represents a dark mode. Interestingly, when both Part 1 and Part 2 are present, we can clearly see the PIT transmission spectrum under the interaction of bright mode and dark mode.

To gain further insights into the physical mechanism behind the observed PIT phenomenon, we analyzed the electric field diagrams. From the diagrams shown in Figure 2b–d, it is apparent that when the incident light is directly irradiated towards the surface of Part 1, the component is excited by the light to generate an electric dipole resonance mode. Conversely, when the incident light is directly irradiated towards the surface of Part 2, it is not excited. However, when both Part 1 and Part 2 are present simultaneously, they interact with each other, leading to the excitation of the dark mode and ultimately resulting in the formation of the PIT phenomenon.

## 3. Results and Discussion

To investigate the impact of the Fermi energy level on the PIT phenomenon, we varied the Fermi energy level of the graphene. In our study, we expressed the Fermi energy level of the individual horizontal and vertical graphene components as *E_f_*_1_ and *E_f_*_2_, respectively. The overall Fermi energy level of the graphene material was expressed as *E_f_*.

**Figure 3:**
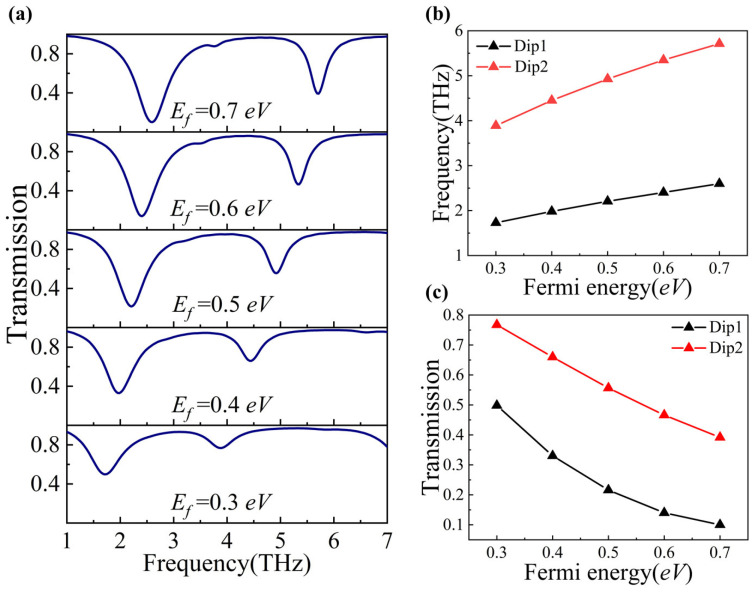
(**a**) Transmission spectra at different *E_f_* values. (**b**) The resonant frequencies of Dip1 and Dip2 with the varied Fermi energy *E_f_*. (**c**) The transmission of Dip1 and Dip2 with the varied Fermi energy *E_f_*.

We increased *E_f_* from 0.3 eV to 0.7 eV in increments of 0.1 eV and observed the changes in the transmission spectra of the H-shaped periodic graphene metasurface. As displayed in Figure 3a, it is evident that with increasing *E_f_*, the resonant frequencies at Dip1 and Dip2 show a blue shift. Figure 3b can also explain and illustrate the blue shift. In addition, the transmission of both Dip1 and Dip2 is significantly decreased, as shown in Figure 3c.

**Figure 4:**
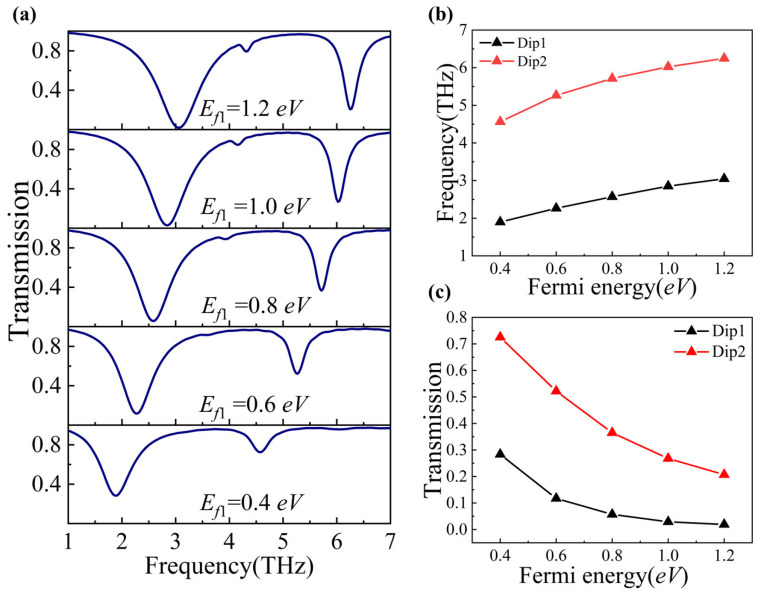
(**a**) Transmission spectra at different *E_f_*_1_ values. (**b**) The resonant frequencies of Dip1 and Dip2 with the varied Fermi energy *E_f_*_1_. (**c**) The transmission of Dip1 and Dip2 with the varied Fermi energy *E_f_*_1_.

**Figure 5:**
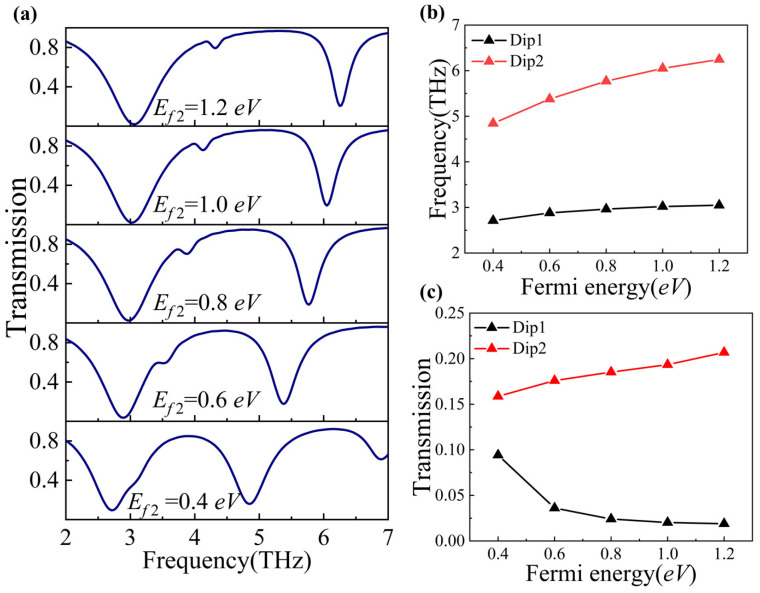
(**a**) Transmission spectra at different *E_f_*_2_ values. (**b**) The resonant frequencies of Dip1 and Dip2 with the varied Fermi energy *E_f_*_2_. (**c**) The transmission of Dip1 and Dip2 with the varied Fermi energy *E_f_*_2_.

We further explored how changes in the Fermi energy levels of the individual horizontal and vertical graphene components (*E_f_*_1_ and *E_f_*_2_, respectively) can modulate the PIT phenomenon. To achieve this, we increased *E_f_*_1_ and *E_f_*_2_ individually from 0.4 eV to 1.2 eV in increments of 0.2 eV. As shown in Figure 4a and Figure 5a, respectively, with an increase in *E_f_*_1_ and *E_f_*_2_, both Dip1 and Dip2 show a blue shift, as also illustrated in Figure 4b and Figure 5b. Meanwhile, the transmission of both Dip1 and Dip2 decreases gradually as we increase *E_f_*_1_, which is shown in Figure 4c. Differently, when we increase the *E_f_*_2_, the transmission of Dip1 decreases, but the transmission of Dip2 increases, as shown in Figure 5c.

Based on our analysis above, it is evident that the resonance frequency and transmission of the PIT phenomenon can be modulated by changing the Fermi energy level of the graphene material as a whole or by changing the Fermi energy levels of Part 1 and Part 2, respectively.

The propagation of SPPs is closely related to the surface structure of metals and nonmetals. Thus, to further explore the PIT phenomenon, we varied some of the shape parameters of the graphene metasurface structure to investigate their impact on the PIT phenomenon.

**Figure 6:**
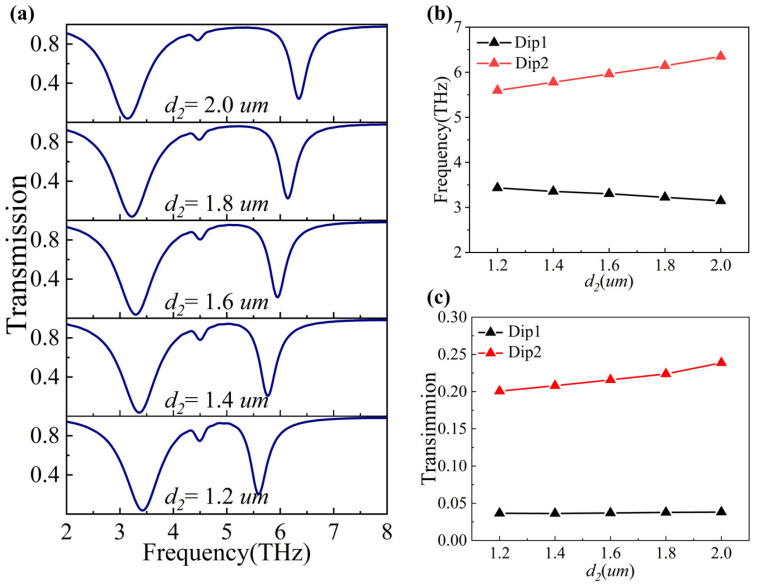
(**a**) Transmission spectra at different *d*_2_ values. (**b**) The resonant frequencies of Dip1 and Dip2 with the varied shape parameter *d*_2_. (**c**) The transmission of Dip1 and Dip2 with the varied shape parameter *d*_2_.

To investigate the effect of changing the shape parameter *d*_2_ on the PIT phenomenon, we increased one of the parameters (*d*_2_) from 1.2 μm to 2.0 μm in increments of 0.2 μm. The changes of the transmission spectra with *d*_2_ are shown in Figure 6a, where it is evident that as *d*_2_ increases, the resonant frequencies at Dip1 show a redshift and the resonant frequencies at Dip2 show a blueshift, as also shown in Figure 6b. Additionally, Dip1’s transmission decreases and then increases at a value of *d*_2_ = 1.4 μm, while Dip2’s transmission increases continually, as shown in Figure 6c. These findings demonstrate that changing the shape parameter *d*_2_ can also allow for the subjective modulation of the resonant frequency and transmission of the PIT phenomena (Dip1 and Dip2).

**Figure 7:**
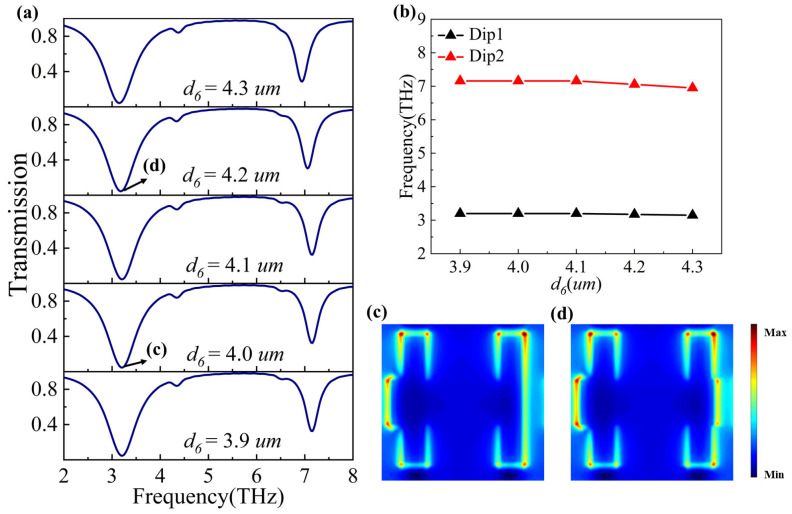
(**a**) Transmission spectra at different *d*_6_ values. (**b**) The resonant frequencies of Dip1 and Dip2 with the varied shape parameter *d*_6_. Figure (**c**,**d**) correspond to the electric field distributions at the points (c) and (d) labeled in Figure (**a**), respectively.

To investigate the effect of varying the shape parameter *d*_6_ on the PIT phenomenon, we keep the coordinates on the left side of *d*_6_ fixed and increase *d*_6_ from 3.9 μm to 4.3 μm in increments of 0.1 μm. As shown in Figure 7a, changes in the transmission spectra are observed as *d*_6_ varies. Interestingly, as depicted in Figure 7b, there is no obvious variation in the resonant frequency when *d*_6_ is in the range of 3.9 μm to 4.1 μm. However, when *d*_6_ is increased from 4.1 μm to 4.3 μm, the resonant frequencies at Dip1 and Dip2 show a redshift. In the graphene metasurface structure shown in Figure 1a, it is apparent that when *d*_6_ lies within the range of 3.9 μm to 4.1 μm, the right-edge coordinates of horizontal graphene lie within the width of the right vertical graphene. Conversely, when *d*_6_ lies within the range of 4.1 μm to 4.3 μm, the right-edge coordinates of horizontal graphene stretch beyond the width of the right vertical graphene. As a result, when *d*_6_ is within the range of 3.35 μm to 4.15 μm, it has a weak impact on the PIT phenomenon, while Dip1 and Dip2 undergo redshift when *d*_6_ falls within the range of greater than 4.15 μm and do not exceed the edge of the unit cell. 

To clarify the physical mechanism behind this phenomenon, we can analyze the electric field distribution map. As can be observed from Figure 7c, there is no electron accumulation on the right edge of the horizontal graphene. Therefore, when *d*_6_ varies within the range of 3.9 μm to 4.1 μm, its electric field distribution remains unaltered, so it does not cause any changes in the PIT phenomenon. In Figure 7d, the electrons are accumulated on the right edge of the horizontal graphene to form an electric quadrupole with the left edge, so Dip1 and Dip2 undergo redshift when *d*_6_ falls within the range of greater than 4.15 μm and does not exceed the edge of the unit cell.

**Figure 8:**
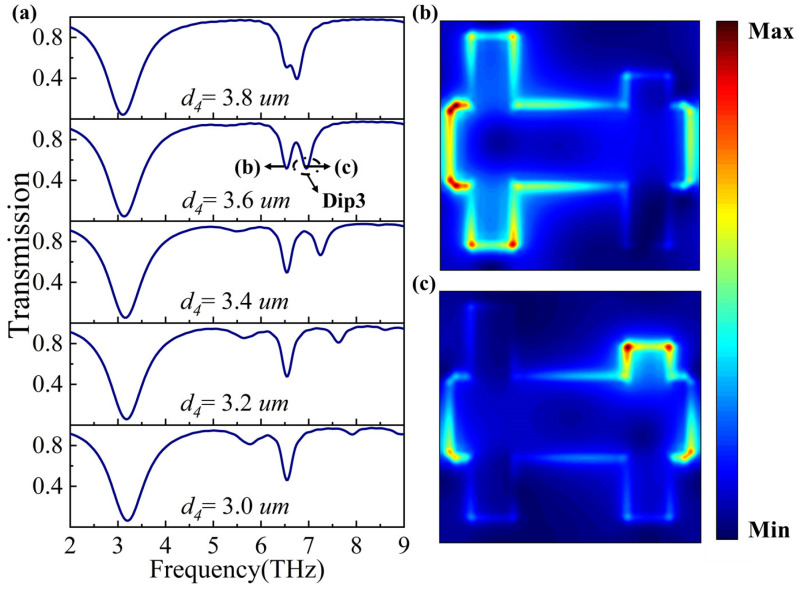
(**a**) Transmission spectra at different *d*_4_ values. Figure (**b**,**c**) correspond to the electric field distributions at points (b) and (c) labeled in figure (**a**), respectively.

Next, we investigate the effect of varying the shape parameter *d*_4_ on the transmission spectra by decreasing it from 3.8 μm to 3.0 μm in decrements of 0.2 μm. As shown in Figure 8a, as *d*_4_ decreases, a new Dip3 gradually emerges at Dip2 and then disappears from the transmission spectra. To understand the physical mechanism behind the generation of Dip3, we plotted the electric field distribution at Dip2 and Dip3. As can be seen in Figure 8b,c, Dip2 is formed due to the interaction of an electric dipole and an electric quadrupole, while two pairs of electric dipoles interact at Dip3, thus leading to the generation of Dip3.

**Figure 9:**
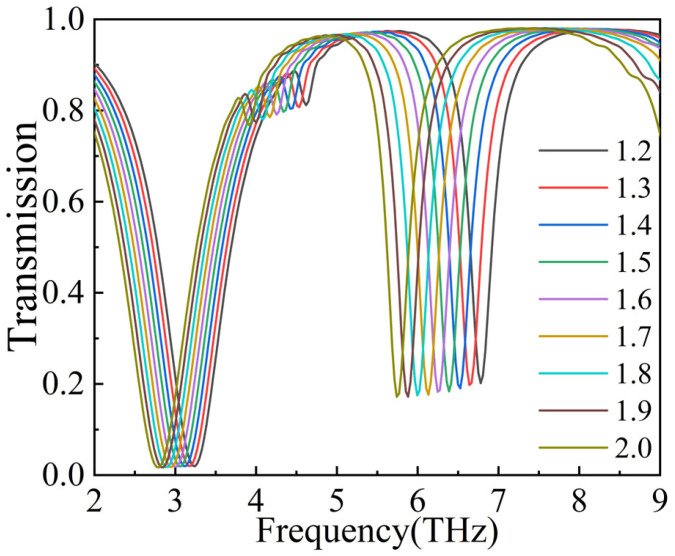
Transmission spectra at different refractive indices for the Fermi energy level of 1.0.

Finally, we evaluated the sensing performance of the proposed graphene metasurface structure. To do this, we increased the refractive index of the outer medium from 1.2 to 2.0 in increments of 0.1, and we analyzed the changes in the transmission spectra, as shown in Figure 9. It is evident that as the refractive index of the outer medium increases, Dip1 and Dip2 undergo redshifts. We then define the sensitivity equation as S = ∆f/∆n and separately explore the sensitivities at Dip1 and Dip2, which are summarized in Table 1. The ∆*f*_1_ and ∆*f*_2_ denote the frequency difference between Dip1 and Dip2, and *S*_1_ and *S*_2_ denote the sensitivity at Dip1 and Dip2, respectively. As can be seen from Table 1, the maximum sensitivity at Dip1 is 0.5612 THz/RIU, while the maximum sensitivity at Dip2 is 1.4028 THz/RIU.

**Figure 10:**
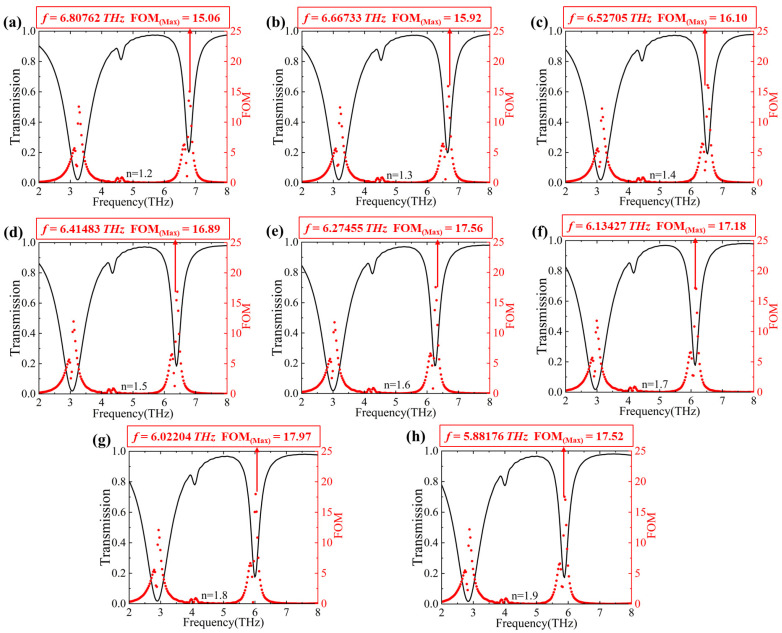
Figure (**a**–**h**) show the FOM values corresponding to refractive indices of 1.2, 1.3, 1.4, 1.5, 1.6, 1.7, 1.8, 1.9, and 2.0, respectively.

By defining the FOM as FOM = ∆T/T∆n [38], where ∆T is the change in transmission and ∆n is the change in refractive index, we calculated the maximum FOM values for the proposed graphene metasurface structure, as shown in Figure 10. From Figure 10, we can find that the maximum FOM values were 15.06, 15.92, 16.10, 16.89, 17.56, 17.18, 17.97, and 17.52 when the refractive index of the outer medium was 1.2, 1.3, 1.4, 1.5, 1.6, 1.7, 1.8, 1.9, and 2.0, respectively.

## 4. Conclusions

In summary, we have presented a novel H-shaped graphene metasurface and investigated its plasmonic properties by using FDTD simulations. Our findings reveal that by changing the Fermi energy level and the shape parameters *d*_2_ and *d*_4_, we can tune the PIT phenomenon. Additionally, altering *d*_4_ enables us to form a new Dip at Dip2. The sensitivity of the proposed graphene metasurface structure can reach up to 1.4028 THz/RIU, and the FOM at the resonance frequency of 6.02204 THz is maximized at 17.97. These results demonstrate the potential of graphene-based plasmonic sensors and provide valuable insights for the development of advanced sensing technologies.

## Figures and Tables

**Table 1:** The sensitivities at Dip1 and Dip2.

∆*f*_1_/THz	*S*_1_	∆*f*_2_/THz	*S*_2_
0.05611	0.5611	0.14028	1.4028
0.05611	0.5611	0.11223	1.1223
0.05611	0.5611	0.14028	1.4028
0.05612	0.5612	0.14028	1.4028
0.05611	0.5611	0.11222	1.1222
0.05611	0.5611	0.14028	1.4028
0.05611	0.5611	0.11223	1.1223
0.02806	0.2806	0.14028	1.4028

## Data Availability

The data that support the findings of this study are available from the corresponding authors upon reasonable request.
